# Anticonvulsant and neuroprotective effects of *Rosa damascena* hydro-alcoholic extract on rat hippocampus

**Published:** 2015

**Authors:** Mansour Homayoun, Masoumeh Seghatoleslam, Mojtaba Pourzaki, Reihaneh Shafieian, Mahmoud Hosseini, Alireza Ebrahimzadeh Bideskan

**Affiliations:** 1 *Department of Anatomy and Cell Biology, School of Medicine, Mashhad University of Medical Sciences, Mashhad, Iran *; 2 *Neurogenic Inflammation Research Center, School of Medicine, Mashhad University of Medical Sciences, Mashhad, Iran*; 3 *Pharmacological Research Center of Medicinal Plants, School of Medicine, Mashhad University of Medical Sciences, Mashhad, Iran*; 4 *Neurocognitive Research Center, School of Medicine, Mashhad University of Medical Sciences, Mashhad, Iran*

**Keywords:** *Rosa damascene*, *Pentylenetetrazol (PTZ)*, *Dark neurons*, *Electrocorticography (ECoG)*, *Hippocampus*

## Abstract

**Objective:**

Previously, analgesic, hypnotic, and anticonvulsant effects have been suggested for *Rosa damascena* (*R. damascena*). In the present study, possible anti-seizure and neuro-protective effects of hydro-alcoholic extract of *R. damascena* has been investigated after inducing seizures in rats by pentylenetetrazole (PTZ).

**Materials and Methods:**

The rats were divided to five groups: (1) Control: received saline, (2) PTZ: 100 mg/kg, i.p., (3) PTZ- Extract 50 mg/kg (PTZ-Ext 50), (4) PTZ- Extract 100 mg/kg (PTZ-Ext 100), and (5) PTZ- Extract 200 mg/kg (PTZ-Ext 200) groups which were treated with 50, 100, and 200 mg/kg respectively of hydro-alcoholic extract of *R. damascena* for one week before PTZ injection. The animals were examined for electrocorticography (ECoG) recording and finally, the brains were removed for histological study.

**Results:**

The hydro-alcoholic extract of *R. damascena* significantly prolonged the latency of seizure attacks and reduced the frequency and amplitude of epileptiform burst discharges induced by PTZ injection. Moreover, all three doses of the extract significantly inhibited production of dark neurons in different regions of the hippocampus in the mentioned animal model.

**Conclusion:**

The present study showed that the hydro-alcoholic extract of *R. damascena* has anticonvulsant and neuroprotective effects. More investigations are needed to be done in order to better understand the responsible compound(s) as well as the possible mechanism(s)**.**

## Introduction

Epilepsy is one of the most common chronic neurological disorders, characterized by repeatedly occurring brain dysfunction called epileptic seizures. A seizure is the physical findings or changes in behavior that occurs after an episode of abnormal electrical activity in the brain (Fisher et al., 2005 ▶). It is caused by a disorganized and sudden electrical activity in a group of neurons that begin firing in an abnormal, excessive, and synchronized manner (Krumholz et al., 2007 ▶). Animal and human studies have provided some evidence that seizure epilepsy causes neural damage and cell death in the hippocampus (Represa et al., 1995 ▶; Arzimanoglou et al., 2002 ▶). There are many potential mechanisms for this cell death, including excitotoxicity from excessive glutamate, release of nitric oxide, increased oxidative stress, and induction of apoptosis (Friedman, 2010 ▶). In addition, seizure significantly increases intracellular Ca^2+^-concentration that is a cellular stress and can be resulted in ultrastructure compaction in the neurons which named dark neurons (Czurkó and Nishino, 1993 ▶; Poirier et al., 2000 ▶; Gallyas et al., 2004 ▶; Ishida et al., 2004 ▶; Kellermayer et al., 2006 ▶). Dark neurons are hyperbasophilic and hyperargyrophilic cells with hyperelectron density properties which appear under specific conditions such as mechanical forces (head injuries or an electric shock), pathological metabolic conditions (hypoglycemia or ischemia), and epilepsy (Zsombok et al., 2005 ▶). It has been shown that generalized seizure produces widespread dark neurons throughout the brain especially in the hippocampus and the pontine reticular formation (Baracskay et al., 2008 ▶).

During the past two centuries, the knowledge of medicinal plants has improved and investigation for new medicines obtained from plants has resulted in the discovery of some clinically useful drugs that have played a key role in the treatment of human diseases (Gorji, 2003 ▶)*. *All over the world,* R. damascena *(*R. damascena*) is a well-known plant for its incredible beauty and aromatic features (Libster, 2002 ▶; Boskabady et al., 2006 ▶). In addition to its fragrance and ornamental use, the flowers are being used for production of rose water and essential oil in several regions of Iran, especially in Kashan (Zargari, 1992 ▶; Libster, 2002 ▶; Rakhshandah et al., 2010 ▶). It has been reported that different extracts of *R. demascena* contain a wide range of compounds including graniol, citrenellol, farnesol, nerol, linalool, citral, eugenol, terpene, myrcene, vitamin C, and bioflavonoid (Boskabady et al., 2006 ▶; Loghmani-Khouzani et al., 2007 ▶; Ulusoy et al., 2009 ▶; Yassa et al., 2009 ▶; Shafei et al., 2010 ▶; Boskabady et al., 2011 ▶). Rose water and essential oil are traditionally used for treatment of stomachache, fever, sore throat, chest pain, ophthalmic problems, menstrual bleeding, breast tenderness, constipation, and other digestive problems (Zargari, 1992 ▶; Libster, 2002 ▶). It is also highly advised for stress adjustment, relaxation and suppression of hypothalamus-pituitary activity (Zargari, 1992 ▶; Ernst et al., 1998 ▶; Rakhshandah and Hosseini, 2006 ▶). Many pharmacological effects such as anti-HIV, anti-microbial, anti-infection, bronchodilatory, antitussive, cardiotonic, cardioaccelerating, and hypoglycemic effects have been reported for this plant by different experimental investigations (Mahmood et al., 1996 ▶; Nascimento et al., 2000 ▶; Biswas et al., 2001 ▶; Aridogan et al., 2002 ▶; Basim and Basim, 2003 ▶; Boskabady et al., 2006 ▶; Gholamhoseinian and Fallah, 2009 ▶; Shafei et al., 2010 ▶). In addition, several studies have indicated that *R. demascena* has some effects on central nervous system including hypnotic, anticonvulsant, anti-depressant, antianxiety, analgesic, and neuroprotective effects (Mahmood et al., 1996 ▶; Rakhshandah and Hosseini, 2006 ▶; Nyeem et al., 2007 ▶; Kheirabadi et al., 2008 ▶; Hajhashemi et al., 2010 ▶; Rakhshandah et al., 2010 ▶; Boskabady et al., 2011 ▶).

Analgesic and hypnotic effects of ethanolic extract of *R. damascena* has revealed by Nyeem et al. (2007) ▶. Moreover, they showed that this plant has suppressor effect on motor activity in rats (Nyeem et al., 2007 ▶). It was also previously shown that essential oil of the plant delays the start of severity epileptic seizures in a PTZ-induced seizure model (Kheirabadi et al., 2008 ▶). Meanwhile, in a study by Hosseini et al. (2011) ▶, it was shown that different extracts of *R. demascena* has an anticonvulsant effect in a mouse model of seizure (Hosseini et al., 2011 ▶). It has been suggested that *R. damascena *has neuroprotective effects in dementia (Awale et al., 2011 ▶). The results of an in vitro study also confirmed the neuroprotective effect of the essential oil and aromatic waters of* R. damascena* (Senol et al., 2011 ▶).

Considering the above mentioned evidence, in this study, we aimed to evaluate the effects of hydro-alcoholic extract of *R*. *damascene* on ECoG criteria (latency, amplitude, duration, and frequency of brain spikes) and neural changes in a pentylenetetrazole (PTZ)-induced seizure model.

## Materials and Methods

**Plant extract**

*R. damascena *shrubs were collected from Kashan (middle part of Iran) in spring and were identified by a botanist. A voucher specimen was preserved in the Herbarium of the School of Pharmacy, Mashhad University of Medical Sciences (Herbarium No: 254-1804-01). The powder of dried flowers (50 g) was extracted in a Soxhlet extractor with ethanol (70%). The resulting extract (yielded 30%) was concentrated under reduced pressure and kept at -4 ˚C until being used (Hosseini et al., 2011 ▶; Hosseini et al., 2012 ▶). Before every injection, the extract was dissolved and stabilized in saline plus a drop of Tween 80 (Rakhshandah and Hosseini, 2006 ▶) then filtered using bacterial filter.

**ECoG recordings and PTZ injection**

The animals were anesthetized using 100 mg/kg ketamine hydrochloride and 20 mg/kg xylazine intraperitoneally (i.p.) and placed in a stereotaxic apparatus. A midline incision was made through the scalp to expose the skull and three holes were created on it (two holes on parietal bones and one in nasal bone in role of a reference). The silver recording and reference electrodes were implanted on the dura mater of the left and right somatosensory cortex and nasal bone respectively and ECoG was recorded using a custom-made deferential ampliﬁer [with band-pass ﬁlters at 0.5–30 kHz, sampling rate 10 kHz, and 0.3–100 Hz (EXT-02 F, NPI, Germany)] and stored by a digital oscilloscope. Recordings were performed for 10 min before and 30 min after intraperitoneally PTZ (100 mg/kg, i.p., Sigma, dissolved in saline) injection (Karimzadeh et al., 2012 ▶). PTZ, a tetrazol derivative, is experimentally used to induce seizures in rodents because of its interaction with the gamma aminobutyric acid (GABA) A receptors complex causes convulsions (Jefferys, 2003 ▶; Ebrahimzadeh Bideskan et al., 2011 ▶; Mohammadpour et al., 2012 ▶; Hosseini et al., 2013 ▶). Latency, amplitude, duration, and frequency of spikes were calculated using AxoScope software.

**Animals and the experimental protocol**

Male Wistar rats, weighing 250-300 g, were housed at 22±2 ˚C and a periodical illumination (ON; 7:00 A.M. to 7:00 P.M.) with food pellets and water available ad libitum. Animal handling and all related procedures were carried out in accordance with Mashhad University of Medical Sciences, Ethical Committee Acts. They were randomly divided to five groups and treated according to the experimental protocol. Group 1 (control group) received saline instead of *R. damascena* extract or PTZ but underwent the surgery procedure and ECoG recording (n = 8). Animals in group 2 (PTZ group) were treated with saline instead of *R. damascena* extract for one week and were then by injected PTZ (100 mg/kg, i.p.) and underwent the surgery procedure and recording (n = 8). Groups 3-5 [PTZ-Ext 50 , PTZ-Ext 100, and PTZ-Ext 200 (n=8 in each)] were treated with 50, 100, and 200 mg/kg of the extract (i.p.), respectively, for one week before PTZ injection and then the ECoG recordings were carried out. 

**Histological studies**

Two hours after finishing ECoG recordings, all of the rats in different groups were injected with an overdose of anesthesia (ketamine, 150 mg/kg, i.p.) and then were perfused through the ascending aorta (the cannula was inserted through the left ventricle) with 100 ml of saline followed by 100 ml fixative solution (4% formaldehyde). Subsequently, the brains were removed and stored in 4% formaldehyde (pH 7.2) for at least 48 h and then processed for dehydration, clearing, and paraffin embedding. Coronal sections of the brains with thickness of 10 μm were cut every 100 μm from 2.3 to 4.3 mm posterior to the bregma (Sadeghian et al., 2012 ▶). The sections were stained with toluidine blue for determining the hippocampal dark neurons and an average of 10 slices were selected. The slides were examined with light microscope (BX51, Japan) at magnification of x40 objective lens (UPlan FI, Japan) and digital photographs were taken from hippocampal regions including CA1, CA2, CA3, and dentate gyrus (DG) of both hemispheres. For quantitative analysis of dark neurons per unit area (NA), unbiased frame, and physical dissector counting rule were carried out (Jafarian et al., 2010 ▶; Mansouri et al., 2013 ▶).

**Statistical analysis**

The data were presented as mean±SEM. The histological data were statistically analyzed using Kruskal Wallis and Mann-Whitney test. However, analysis of variance (ANOVA) followed by Tukey’s post hoc test was carried out for comparing of ECoG recording criteria. Significance was established when the probability values were less than or equal to 0.05.

## Results

**The effect of** **hydro-alcoholic extract of** ***R*** **.** ***damascena*** **on PTZ-induced seizures**

**Figure 1:**
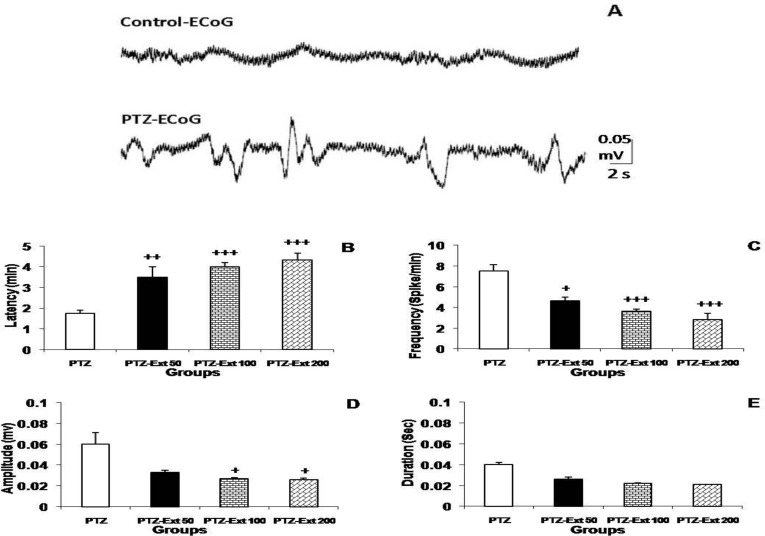
The effect of hydro-alcoholic extract of *R. damascena* on epileptic activities induced by injection of pentylenetetrazol (PTZ) in rats. (A) Sample of epileptiform burst discharges recorded by electrocorticogram (ECoG) in PTZ group compared with control group. The latency time of spikes in PTZ-Ext 50, 100, and 200 groups was significantly longer than PTZ group. The frequency of burst discharges in PTZ-Ext 50, 100, and 200 groups was significantly lower than PTZ group. The amplitude of spikes in PTZ-Ext 100 and 200 groups was significantly lower than PTZ group. There were no significant differences of the mean duration of burst discharges between the extract pre-treated animals and PTZ group. +p<0.05 ++p<0.01and +++p<0.001 compared to PTZ group.

Administration of 100 mg/kg of PTZ induced a tonic-clonic seizure in all anesthetized rats. The seizure discharges which were observed during seizure attacks are shown in Figure 1A. The latency to the first discharge in PTZ group was 1.75±0.14 min. Pretreatment with 50-200 mg/kg of the extract postponed the starting of the discharges to 3.5±0.50, 4±0.20, and 4.33±0.33 min, respectively (p<0.01- p<0.001; Figure 1B). The frequency of the seizure discharges in PTZ group was 7.5±0.64/min. Pretreatment with 50, 100, and 200 mg/kg of the extract before PTZ decreased the frequencies to 4.66±0.33, 3.62±0.23, and 2.83±0.6 /min, respectively (p<0.05, p<0.001, and p<0.001; Figure 1C). The results also showed that 100 and 200 mg/kg but not 50 mg/kg of the extract decreased the amplitude of the epileptiform potentials (p<0.05; Figure 1D). There were no significant differences of the mean duration of burst discharges between the extract pre-treated animals and PTZ group (Figure 1E).

**The effect of hydro-alcoholic extract of** ***R*** **.** ***damascena*** **on production of dark neurons**

**Figure 2:**
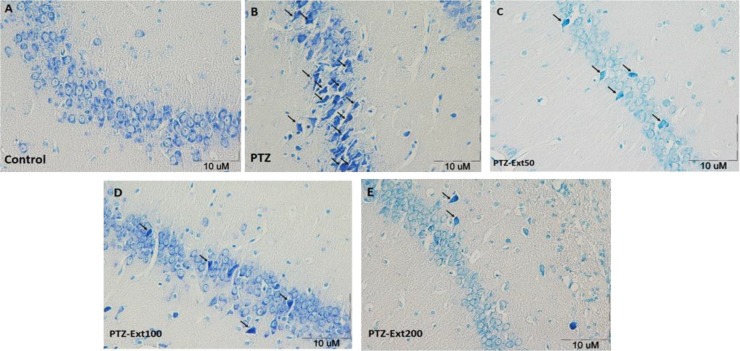
Light-microscopic appearance of toluidine blue stained normal pyramidal cells and dark neurons in coronal sections of the hippocampus in different groups. A section from control group, showing normal small pyramidal cells (A). A section of PTZ group, showing dark neurons (arrows) among pyramidal cells (B). A section of PTZ-Ext 50 group, showing dark neurons (arrows) among pyramidal cells (C). A section of PTZ-Ext 100 group, showing dark neurons (arrows) among pyramidal cells (D). A section of PTZ-Ext 200 group, showing dark neurons (arrows) among pyramidal cells (E).

**Figure 3:**
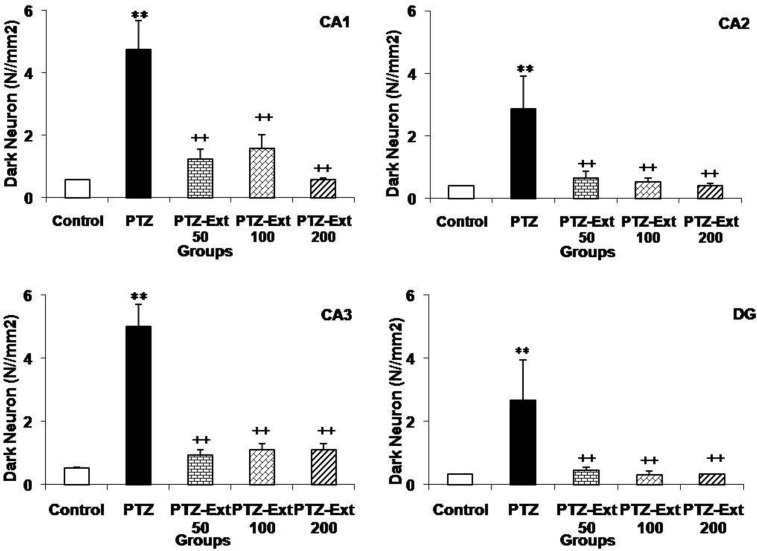
The inhibitory effect of hydro-alcoholic extract of *R*. *damascena* on production of dark neurons. *R. damascena* extract at doses of 50, 100, and 200 mg/kg inhibited production of dark neurons in the hippocampal CA1, CA2, CA3, and DG areas in rats after induction of seizure attacks by intraperitoneal pentylenetetrazol (PTZ) injection. Dark neurons values in CA1, CA2, CA3, and DG regions of PTZ group were significantly more than control groups, while *R. damascena* extract reduced significantly the number of dark neurons in all areas in PTZ group. ^ **^p<0.01 compared to control group, ^ ++^p<0.01 compared to PTZ group

Dark neurons were characterized by cytoplasmic and nuclear condensation and neuronal shrinkage in hippocampal formation [Figure 2 (B, C, D, E)]). After injection of PTZ, numerical density of dark neurons in the hippocampal CA1, CA2, CA3, and DG areas of the PTZ group rats was significantly increased in comparison to the control group (p< 0.01; Figure 3).

The mean values for dark neurons in CA1, CA2, CA3, and DG areas of control group were 0.57, 0.41, 0.52, and 0.33 per unit area (N/mm^2^), respectively, whereas the mean values for dark neurons in CA1, CA2, CA3, and DG areas of PTZ group were 4.75, 2.86, 5, and 2.66 N/mm^2^, respectively. Comparison of different parts of the hippocampus revealed that numerical densities of dark neurons in the CA1 and CA3 areas were more than CA2 and DG. Administration of *R*. *damascena *extract at doses of 50, 100, and 200 mg/kg significantly prevented production of dark neurons by PTZ injection in all regions and reduced the mean number of dark neurons (p< 0.01; Figure 3). Injection of 50 mg/kg of *R*. *damascena *extract reduced the mean values of dark neurons in CA1, CA2, CA3, and DG areas to 1.23, 0.66, 0.93 and 0.46 N/mm^2^, respectively. Administration of *R*. *damascena *extract at concentration of 100 mg/kg reduced the mean values of dark neurons in CA1, CA2, CA3, and DG areas to 1.58, 0.53, 1.11, and 0.3 N/mm^2^, respectively. Injection of 200 mg/kg of *R*. *damascena *extract also reduced the mean values of dark neurons in CA1, CA2, CA3, and DG areas to 0.58, 0.41, 1.11, and 0.33 N/mm^2^, respectively. There was no significant difference between all three groups (50, 100, and 200 mg/kg) in the number of dark neurons of CA1, CA2, CA3 and DG areas.

## Discussion

The findings of this study demonstrated that administration of hydro-alcoholic extract of *R. damascena* in different doses signiﬁcantly attenuated the latent period to the beginning of the convulsion and reduced the amplitude and frequency of epileptiform burst discharges induced by PTZ injection in rats but did not affect the duration of these discharges. Moreover, the data showed a significant reduction in the number of dark neurons in the hippocampus in mentioned animal models.

Previous studies have shown that *R. damascena* has extensive effects on central nervous system (Rakhshandah and Hosseini, 2006 ▶). In line with our results, anticonvulsant effect of this plant has been reported in different animal seizure models and epileptic humans (Ashrafzadeh et al., 2007 ▶; Kheirabadi et al., 2008 ▶; Ramezani et al., 2008 ▶; Hosseini et al., 2011 ▶). Kheirabadi et al. used the *R. damascena* essence on a chronic model of PTZ-induced seizure. In their study prior to PTZ injections, the experimental groups of rats received 500, 750, and 1000 mg/kg essential oil of *R. damascena* and during the experimental period the epileptiform, and behaviors of all rats were evaluated before and after essential injections. The results showed that the *Rosa* essential oil increased the seizure latency as well as reduced the severity of seizures in a dose-dependent manner (Kheirabadi et al., 2008 ▶). In addition, in the other study, injection of essential oil before amygdale electrical kindling reduced appearance of different stages of seizure and reduced the after discharge duration (Ramezani et al., 2008 ▶). The antiepileptic effect of the essential oil of *R. damascena* in children with refractory seizures has also been suggested (Ashrafzadeh et al., 2007 ▶). Another study by Hosseini et al. revealed anticonvulsant effects of different extracts of *R. demascena* on PTZ-induced seizures (Hosseini et al., 2011 ▶). Pretreated of the PTZ-injected mice with different doses of aqueous, ethanolic, and chloroformic extracts of *R. demascena* and showed a significant increase in latencies of minimal clonic seizures and generalized tonic-clonic seizures. In the present study, using ECoG method, the anticonvulsant effects of hydro-alcoholic extract of the flower was confirmed. However, in spite of Kheirabadi and Ramezani´s results, we demonstrated that the duration of epileptiform burst discharges did not change significantly in extract groups compared to PTZ group. It seems that the most convincing explanation is difference in the concentration of anticonvulsant components (flavonoieds, geraniol, and eugenol) in essential oil and extracts of *R. demascena. *However, further studies are needed to clarify this issue.

Seizure has been shown to induce neuronal structure impairments in hippocampal formation, including the hippocampus and dentate gyrus (Kohl et al., 2011 ▶). Thus, memory impairment is one of the most important defects of epileptic patients (Kohl et al., 2011 ▶). Considering these facts, continuing researches highlight the value of new chemical or natural anticonvulsant compounds with neuroprotective effects. The results of present study showed for the first time that the hydro-alcoholic extract of *R. demascena* has potential neuroprotective effects in seizure which was presented by prevention of appearance of dark neurons in several regions of hippocampal formation. Dark neurons have been reported to be appeared in various pathological conditions including epilepsy, stroke, hypoglycemia, aging, and spreading depression phenomenon (Czurkó and Nishino, 1993 ▶; Ishida et al., 2004 ▶; Kherani and Auer, 2008). It is also suggested that dark neuron is formed in stressful conditions such as mechanical trauma to the brain prior to fixation (Ooigawa et al., 2006 ▶). It has also been shown that seizures may lead to morphological changes such as production of dark neurons in brain tissue (Toth et al., 1998 ▶; Karimzadeh et al., 2012 ▶). Disturbances in ion gradient and increased activity of excitatory neurotransmitter systems such as glutamate and aspartate have been suggested as the main contributors in dark neuron production (Kherani et al., 2008 ▶). It is also suggested that the brain tissues oxidative damages due to free radicals, glutamate, and aspartate have a role in the production of dark neurons (Ankarcrona et al., 1996 ▶; Kherani and Auer, 2008 ▶). On the other hand, the antioxidant effect of *R. damascene* and its inhibitory effect on lipid oxidation have also been reported (Kumar et al., 2009 ▶). A protective effect against brain tissues oxidative damages of *R. demascena* was also confirmed in our previous study (Mohammadpour et al., 2014 ▶) which might be considered as an explanation for the neuroprotective effect which was seen in the present study, however, it needs to be more investigated in the future. The compound(s) responsible for anticonvulsant effect of *R. damascene* is (are) unknown and was not investigated in the present study but it has been previously suggested that the flavonoid compounds of this plant may be involved in this effect (Rakotonirina et al., 2001 ▶). The involvement of GABA neurotransmission in convulsion, sleep, analgesia, and locomotors activity is obvious, and since flavonoids can act on GABAergic system in the brain (Kheirabadi et al., 2008 ▶), it might be deduced that this compounds which exists in *R. damascene* may have interaction with GABA system. The other plant extracts which containing flavonoid compounds have been reported to have both anticonvulsant and antioxidative effects in PTZ-induced seizure model (Hosseini et al., 2013 ▶; Hosseini et al., 2014 ▶). Therefore, the neuroprotective effects of the extract which were observed in the present study might be at least in part due to the flavonoid compounds. Other components of *R*.* damascena* such as geraniol and eugenol have been shown to have antiepileptic effect (Wie et al., 1997 ▶) which may contribute to the results of present study. 

The results of the current study using ECoG method confirmed the anticonvulsant effects of hydro-alcoholic extract of R. damascena which was accompanied with the protection against neural damage. These results support the traditional belief about the beneficial effect of R. damascena on the nervous system. Further studies are required for better understanding of the exact responsible mechanism(s) and compound(s).
